# Valorization of hydro-distillate of fruit peels of *Citrus paradisi* macfad. Cultivar. Foster: Chemical profiling, antioxidant evaluation and in vitro and in silico enzyme inhibition studies

**DOI:** 10.1016/j.heliyon.2024.e36226

**Published:** 2024-08-21

**Authors:** Rameen Sajid, Zaheer Abbas, Mamona Nazir, Muhammad Saleem, Naheed Riaz, Muhammad Imran Tousif, Saba Tauseef, Gokhan Zengin, Abdullahi Ibrahim Uba, Abdullah Ijaz Hussain, Muhammad Shaiq Ali, Abeer Hashem, Khalid F. Almutairi, Graciela Dolores Avila-Quezada, Elsayed Fathi Abd_Allah

**Affiliations:** a Institute of Chemistry, Baghdad-ul-Jadeed Campus, The Islamia University of Bahawalpur, 63100, Bahawalpur, Pakistan; b Department of Botany, Division of Science and Technology, University of Education Lahore, Pakistan; c Department of Chemistry, Government Sadiq College Women University Bahawalpur, 63100 Bahawalpur, Pakistan; d Department of Chemistry, Division of Science and Technology, University of Education Lahore, Pakistan; e Dr. Panjwani Center for Molecular Medicine and Drug Research., International Center for Chemical and Biological Sciences, University of Karachi, Karachi, Pakistan; f Department of Biology, Science Faculty, Selcuk University, 42130 Konya, Turkey; g Department of Molecular Biology and Genetics, Istanbul AREL University, 34537 Istanbul, Turkey; h Department of Chemistry, GC University Faisalabad, Pakistan; i International Center of Chemical and Biological Sciences, University of Karachi, 75270 Karachi, Pakistan; j Botany and Microbiology Department, College of Science, King Saud University, P.O. Box. 2460, Riyadh 11451, Saudi Arabia; k Facultad de Ciencias Agrotecnológicas, Universidad Autónoma de Chihuahua, 31350, Chihuahua, Chihuahua, Mexico; l Plant Production Department, College of Food and Agricultural Sciences, King Saud University, P.O. Box. 2460, Riyadh 11451, Saudi Arabia

**Keywords:** *Citrus paradisi* cultivar foster, Essential oil, GC-MS analysis, Antioxidant, Enzyme inhibition, *In silico* studies

## Abstract

The major commercial crops in Pakistan are citrus fruit trees, which are farmed extensively and serve as the country's principal source of foreign exchange. A local citrus plant, *Citrus paradisi*, variety Foster is famous for its valuable fruit and fruit juice, however, tons of peels of this fruit are thrown as waste, which otherwise can be utilized in formulating nutraceutical and cosmetics. In the present study, essential oil of fruit peels was obtained through hydro-distillation, which was then analyzed through GC-MS analysis and studied for its antioxidant and enzyme inhibition potential. GCMS analysis revealed the presence of several components; major were found to be limonene, α-terpineol, caryophyllene, *δ*-amorphene, elemol, γ-eudesoml, nootkatone and di-isooctyl phthalate. Although, the oil showed weak free radical inhibition, it was potentially active in CUPRAC, FRAP, phosphomolybdenum and metal chelating antioxidant assays. The oil also exhibited anti-glucosidase, anti-amylase activities and also exhibited potent inhibition of the enzyme tyrosinase, which makes it strong candidate for nutraceuticals and skin care products. The docking studies also substantiate our results and caryophyllene, γ-eudesoml and nootkatone showed good binding affinity α-glucosidase and α-amylase and all tested compounds showed the higher binding affinity towards the enzyme tyrosinase.

## 1 Introduction

The Genus *Citrus* of the plant family Rutaceae, is one among the main fruit crops in tropical, subtropical and temperate regions around the globe [1,2]. Striking feature of the citrus plants is that, hybrid varieties are easily be produced by cross breeding among different species [3], which results into increased production of citrus fruits with various fragrance, flavor and types. The most common citrus fruits are oranges, grapefruits, mandarins, lemons and limes, which are popular due to their flavors, nutritional and health benefits and thus are the main industrialized crops all over the world [2,4]. Recent statistical analysis revealed that the global business of citrus was estimated as USD 7601.7 million in 2023, which is predicted to increase 10437.1 million in 2030 [5]. Citrus plants are aromatic due to their volatile oil contents [6], besides they produce bioactive secondary metabolites that can combat against disease and promote human health. Citrus fruits are rich in vitamin C, folic acid, potassium and pectin, in fact vitamin C contents are higher in fruit peels as compared to juice [7,8].

Several studies have proved that citrus peels and their extracts exhibit potent pharmacological activities and health benefits due to their bioactive contents and antioxidant properties [9]. In addition to antioxidant phenolics and flavonoids, citrus peels are rich source of volatile oils with higher limonene and vitamin C contents. Published data shows that appropriate intake of antioxidants improves the immune system and thus reduces the severity and duration of colds, flus, and other viruses [10]; it makes citrus fruits a precious food crop.

Several studies have been conducted on extraction, identification and bioactivities of essential oils of citrus fruit peels. For example peel essential oil of *C. aurantifolia* has been reported to possess *in vivo* cholesterol lowering properties [11]. Matsuura et al. studied tyrosinase inhibitory activities of several citrus fruit peel essential oils and found significant potential associated to these oils [12]. Sweet orange, bergamot and lemon fruit peel essential oils showed significant larvicidal activity [13]. Lemon fruit peel essential oil has also been reported to exhibit antioxidant and antimicrobial activities [14]. Orange fruit peel essential oil inhibited the growth of various food spoiling and other pathogenic bacteria [[15], [16], [17]]. Hydro-distilled essential oils of fruit peels of mandarin were found antioxidant which also showed antimicrobial activities against Leuconostoc mesenteroides, *Escherichia coli* and Lactobacillus plantarum [18]. Another report describes that essential oil of fruit peel of *C. reticulate* exhibit antibacterial and wound healing properties [19]. DPPH* inhibitory activities of fruit peel essential oils of C. reticulate, C. paradisi and C. lemon have been reported along with antimicrobial activities against Bacillus subtilis, Penicillium chrysogenum, Fusarium moniliforme, Aspergillus niger, Aspergillus flavus, *Saccharomyces cerevisiae*) and pathogenic microorganisms (*Escherichia coli*, Salmonella abony, *Staphylococcus aureus*, *Pseudomonas aeruginosa*, Candida albicans), and thus essential oils of these fruits can be used in bio-preservation strategies [20,21]. Interestingly, citrus peel essential oils also increased the shelf-life of strawberries and thus played important role in food preservation [22].

*Citrus paradisi* (common name grapefruit) is a hybrid of two *Citrus* species; *C. sinensis* and *C. maxima* or *Citrus grandis* Osbeck [23,24]. It is native to island of Barbados in West Indies, but also widely grown in different parts of Asia, America, Israel, Cuba, Argentina, and South Africa [25,26]. Two common varieties of grapefruit are available depending upon the color of the pulp; the white and redblush or foster pink.

In addition to its importance as food crop, *C. paradisi* is medicinally very important natural source. Its juice decreases diastolic arterial pressure and systolic arterial pressure both in normotensive and hypertensive subjects [27]. It is also effective in gastric problems and exhibit antiulcer properties due its antioxidant flavonoids. Its flavonoids are also reported to possess antibacterial, anti-helicobacter pylori activity showed cytoprotection against injury [28,29]. Grapefruit peel essential oils exhibited antibacterial properties against Salmonella *parathypi*, Vibrio vulnificus and Seratialique faciens [30], and several other pathogenic bacteria and fungi [31]. Some studies revealed that peels of grapefruit are used in controlling diabetes and hypertension [32]. Grapefruit essential oil also exerted inhibitory effects on the proliferation of HepG2 liver cancer cells and HCT116 colon cancer cells [33].

Although citrus fruit plants are the major commercial crops in Pakistan which are grown at wide area and the export valued about 166 million USD [34], the research on local variety Foster of *C. paradisi* is only scanty, which otherwise may provide a potential source to develop nutraceuticals and cosmetic ingredients. Despite of being a tremendous source of nutrients, chemicals, minerals and its cholesterol lowering property, yet its peels are thought to be as waste in local market. Further, fruit peels or its essential oils are not much explored locally for its medicinal values. Fewer studies disclosed the yield of essential oil from *C. paradisi* is ∼3.9 % comprising of aliphatic hydrocarbons, alcohols, aldehydes, ketones, esters and terpenoids [35]. Limonene the main components of various oils have anti-proliferative action. Cell proliferation was affected by limonene in a biphasic manner; an increase in peroxidase and catalase activity was linked to a reduction in H_2_O_2_ levels. Additionally, limonene shielded the cells against the exogenous injection of H_2_O_2_-induced oxidative damage [36]. Furthermore, the synthesis of cytokines is modulated by limonene and is able to alter signalling pathways associated with a number of illnesses [37].

In the present study, hydro-distilled essential oil of locally grown grapefruit peels was analyzed through GC-MS and was evaluated for its free radical inhibition (DPPH*, ABTS*^+^), metal reducing (FRAP, CUPRAC), metal chelating and enzyme (AChE, BChE, tyrosinase, α-glucosidase and α-amylase) inhibition activities.

## 2 Experimental

### 2.1 Plant material

The fresh fruits of *C. paradisi* (Grapefruit-Foster) were purchased from local market in Bahawalpur, which were authenticated by Dr. Farrukh Nisar, plant taxonomist in the department of Biochemistry, Cholistan University Bahawalpur, Pakistan. The fruits were peeled and pulp and inner peel skin was fully removed from the peels.

### 2.2 Extraction of the essential oil

The peels (2.0 kg) of *C. paradisi* were cut into small pieces and were subjected to hydro-distillation for approximately 3 h. A recirculating chiller (0 °C) was used for condensation of distilled vapors. The distillate obtained were separated by separating funnel and moisture was removed from the pale yellow layer of essential oil by adding anhydrous sodium sulphate and then stored in vials at 4 °C for further analysis and biological studies.

### 2.3 GC-MS analysis

A GC TRACE-1300 gas chromatograph connected to an MS, ISQ, and auto sampler AI-1310 was used to analyze the essential oil. The instrument used was a capillary column TR-35 MS GC Column 30mx.25mmIDx.25μm. The analysis used an injector temperature of 250 °C, and the column temperature was first programmed to be 50 °C for 5 min, then to be 5 °C/min to 140 °C, then 7 °C/min to 275 °C, and finally to be held for 10 min. The temperature of the transfer line was 250 °C, and the temperature of the ion source was 200 °C. The sample delay period started at 3.5 min. Split less injection was utilized, and the carrier gas used was helium 99.9992 % with a gas flow rate of 1.5 ml/min. Mass spectra were obtained after a 1-μl volume was injected and 70 electron volts of ionization energy were used. The NIST (National Institute of Standards and Technology) Mass spectral search and library system was utilized by the Xcalibur software data system to analyses the obtained mass spectra. The NIST/EPA/NIH main library was the library that was used to match data.

### 2.4 Antioxidant activities assays

The essential oil's antioxidant activity was estimated by following pre-established protocols (For details see supplementary file). For FRAP, ABTS*^+^, DPPH*, CUPRAC, and total antioxidant capacity, trolox equivalent was utilized as the standard; for metal chelating assays, ethylene diamine tetraacetic acid (EDTA) was the standard [38,39].

### 2.5 Enzyme inhibition assays

The α-amylase, α-glucosidase, BChE, tyrosinase, and AChE enzyme inhibitory assays were conducted using previously published methods. The inhibitory activity of α-amylase and α-glucosidase was measured using acarbose (mmol ACAE/g extract) as the standard, the inhibitory activity of AChE and BChE was measured using galantamine (mg GALAE/g extract), and the inhibitory activity of tyrosinase was measured using kojic acid (mmol KAE/g extract) [40,41]. For antioxidant and enzyme inhibitory assays, the sample solutions were carefully formulated in ethanol at a concentration of 2 mg/mL. All chemicals were freshly prepared, with special attention to keeping them in an ice bath for the upcoming use (For details see supplementary file).

### 2.6 Docking analysis

Molecular docking studies can be very helpful for learning about possible drugs' selectivity and steric properties, which can be used to anticipate their locations of binding for potential inhibition. and improvement of potential therapeutic drugs [42]. The 3D sdf structures of five compounds were retrieved from PubChem [43]. The structures were converted to pdb format and energy was minimized prior to docking through Chem3D Ultra 16.0. the three protein structures were retrieved from the Protein Data Bank (PDB) database [44]. In order to identify the potential of drug-target interaction, Autodock software (version 4.2) was used to perform molecular docking on five compounds with α-amylase, α-glucosidase and tyrosinase using the Lamarckian genetic method [45]. The receptor proteins remained rigid, while the ligands were made flexible in order to move and investigate the most likely binding postures. The docking was performed in a similar fashion reported previously [46]. The resulting binding poses were visualized using Discovery Studio Visualizer 3.5 [47]. The interactions were investigated in terms of binding energy (Kcal/mol), Ki (Inhibition constant) value (M), and the hydrogen bonds formed and interaction patterns observed within residues of protein and ligand.

## 3 Results and discussion

**Fig. 1:**
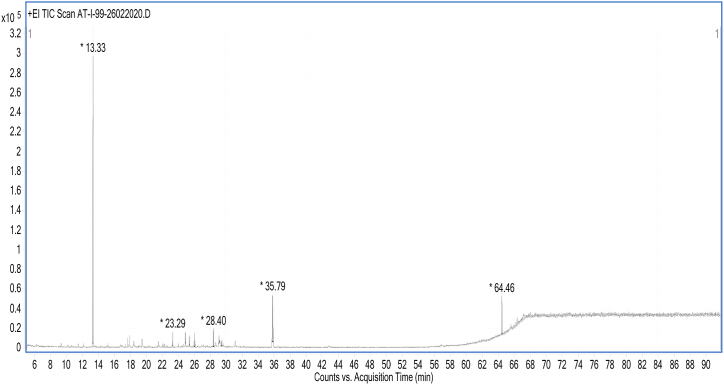
Gas chromatogram of *Citrus paradisi* cultivar Foster peel essential oil (HDEO).

Pale yellow dried hydro-distilled essential oil (HDEO) of fruit peels of *Citrus paradisi* cultivar Foster was obtained in 1.18 % (w/w) yield, which exhibited pleasant citrus smell. A complex chromatogram of HDEO was obtained as result of gas chromatography (Fig. 1), however, 12 components could be identified through their retention time and fragmentation patterns in their respective mass spectra. The identified metabolites of HDEO along with their retention time and percentage area are shown in Table 1. 97.09 % of the total HDEO comprised of only 08 metabolites (Table 1), while others were found in trace amounts. Most of the constituents of HDEO were found as monoterpene hydrocarbons, aliphatic aldehydes, sesquiterpenes, esters, acids, alcohols, ketones, and other oxygenated compounds with limonene (53.81 %) and nootkatone (22.12 %) as major components. Okunowo et al. also published a report on hydro-distillation of grapefruit peel essential oil with limonene as major component as reported by adebisi et al. [48]; however, they did not observe nootkatone [31], while other reports revealed nootkatone as one among the major components of *C. paradisi* fruit peel essential oil [21,23,[49], [50], [51]] with varying % age.

**Table 1:** Constituents of *Citrus paradisi* cultivar Foster fruit peel hydro-distilled essential oil (HDEO).

No.	RT(min)	RI	Mass fragments (*m/z*)	Molecular Formula	Compound	Mode of identification	Peak area (%)
1	13.33	1029	136, 121, 107, 93, 79, 68, 53, 41	C_10_H_16_	Limonene	RT, RI,MS	53.81
2	17.67	1189	154, 121, 93, 81, 59, 43	C_10_H_18_O	α-Terpineol	RT, RI,MS	Tr
3	17.89	1497	112, 95, 82, 68, 57, 43	C_10_H_20_O	Decanal	RT, RI,MS	Tr
4	19.46	–	111, 97, 83, 70, 69, 55, 43, 41	C_10_H_22_O	1-Decanol	RT, MS	Tr
5	21.50	–	172, 129, 83, 73, 69, 60, 56, 43	C_10_H_20_O_2_	n-Decanoic acid	RT, MS	Tr
6	23.29	1583	189, 175, 161, 147, 133, 120, 105, 91, 79, 69, 55, 41	C_15_H_24_	Caryophyllene	RT, RI,MS	2.37
7	24.88	–	206, 191, 175, 163, 147, 128, 119, 107, 91, 77, 60, 57, 44, 41	C_14_H_22_O	2,4-Di-tert butyl phenol	RT, MS	Tr
8	25.38	1442	204, 189, 161, 143, 134, 129, 119, 105, 91, 77, 67, 55, 41	C_15_H_24_	*δ*-Amorphene	RT, RI,MS	1.45
9	25.99	1550	189, 161, 147,134, 119, 107, 93, 79, 67, 59, 55, 43	C_15_H_26_O	Elemol	RT, RI,MS	2.67
10	28.40	–	222, 204, 189, 175, 161, 147, 133, 119, 107, 91, 79, 67, 59, 55, 41	C_15_H_26_O	γ-Eudesoml	RT, MS	5.33
11	35.79	2563	218, 203, 190, 175, 161, 147, 133, 121, 105, 91, 79, 67, 53, 51	C_15_H_22_O	Nootkatone	RT, RI,MS	22.12
12	64.46	–	279, 207, 190, 167, 149, 113, 83, 71, 57, 43	C_24_H_38_O_4_	Di-iso-octyl phthalate	RT, MS	7.39
			Total identified %				97.09
	Varies		–	–	Others		2.91

RT = Retention time (compounds were listed in order of elution from an HP-5MS column); RI = Retention indices, Retention indices relative to C9–C24 n-alkanes on the HP-5MS column; Tr = Trace amount t = trace (<0.05 %); MS = identification based on comparison of mass.

Additional substances found in HDEO included aliphatic aldehydes like decanal, alcohols like α-terpineol and 1-decanol, and trace amounts of acids like *n*-decanoic acid. Prominent components included caryophyllene (2.37 %), δ-amorphene (1.45 %), di-iso-octyl phthalate (7.39 %), elemol (2.67 %), γ-eudesmol (5.33 %), and 2,4-DTBP (2,4-di-tert-butylphenol) (1.95 %). This source has yielded the first report of 2,4-di-tert-butylphenol in the current study. When comparing our sample of *C. paradisi* to those published in the literature by Okunowo et al., the percentages of caryophyllene and phthalate were higher, mounting up to 1.88 % and 0.54 %, respectively [31]. Previous studies have noted that during different developmental stages of fruit ripening, the content and composite function of oil can even vary within the same genus [16]. Using a similar method, Karioti et al. reported that the main compounds in the Nigerian mandarin leaf oil were γ-terpinene and linalool, while limonene was the main constituent in the peel oils of both grapefruit and Nigerian mandarin [52]. Additionally, literature reports that the essential oils of *C. paradisi* had a higher percentage of the limonene component [53], and consequently, the published data supported our findings. In contrast to its previously studied leaf oil (11.37 %) from Nigeria, the percentage of limonene component in the peel oil of *C. paradisi* was higher (53.881 %) in the recently investigated peel oil [52]. and this variation must be because of environmental changes.

### 3.1 Antioxidant activities of the HDEO

Fruit peel hydro-distilled essential oil (HDEO) of *C. paradisi* was investigated for its antioxidant activities in various assays including DPPH* and ABTS*^+^ free radical scavenging, metal reducing, metal chelating and Phosphomolybdenum assays. Against DPPH* free radical, HDEO was found inactive, however, it showed mild (3.98 ± 0.36 mgTE/g extract) scavenging activity against ABTS*^+^. Literature reports revealed that other citrus fruit essential oils exhibited free radical scavenging activities [[54], [55], [56], [57]], however, grapefruit essential oil is reported almost inactive against DPPH* free radical [58]. Therefore, literature reports substantiated our results, while the activity difference could be attributed to the absence of phenolic components in HDEO. In cupric (CUPRAC) and ferric (FRAP) reducing capacity assays, HDEO exhibited significant activity with the values of 45.15 ± 1.59 and 24.33 ± 0.18 mgTE/g extract, respectively (Table 2). Gargouri et al. have reported similar results where 60 % ferric reducing power of *C. paradisi* essential oil was observed, whereas, in another study, the essential oils of *C. sinensis* [59,60] and *C. limon* [61] have shown mild cupric reducing power. Since no single component of HDEO has been reported in literature to show metal reducing capacity, the activity of HDEO could be attributed to synergic effect of its various components. In Phosphomolybdenum assay, a significant total antioxidant capacity has been observed (0.73 ± 0.08 mmolTE/g) for HDEO which is fully supported by the literature values [59,62]. Iron chelating activity of HDEO was also measured, where it showed significant chelating value of 1.15 ± 0.10 mg EDTAE/g extract (Table 2), which has also been supported by the previously published data [63,64]. According to a recent study, the essential oil extracted from the peel of *Citrus reticulata* Blanco demonstrated potent antioxidant properties, including the ability to scavenge DPPH* and ABTS*^+^ radicals, as well as H_2_O_2_ and ferric reducing antioxidant capacity and prevent lipid peroxidation [65]. In another investigation, the *citrus limetta risso's* limonene-rich essential oil demonstrated its ability to quench radicals using DPPH radical scavenging (11.35 ± 0.51 μg/mL) and ABTS scavenging (10.36 ± 0.55 μg/mL) [66].

**Table 2:** Antioxidant and enzyme inhibitory activities of the of *Citrus paradisi* peels hydro-distilled essential oil (HDEO).

Sample code	Antioxidant activity					
HDEO	Radical scavenging assays		Reducing power assays		Total antioxidant capacity	Ferrous ion chelation
	DPPH^a^ (mgTE/g extract)^a^	ABTS^a^^+^ (mgTE/g extract)^a^	CUPRAC (mgTE/g extract)^a^	FRAP (mgTE/g extract)^a^	Phosphomolybdenum (mmolTE/g extract)^a^	Metal chelating (mgEDTAE/g extract)^a^
	na	3.98 ± 0.36	45.15 ± 1.59	24.33 ± 0.18	0.73 ± 0.08	1.15 ± 0.10
	**Enzyme inhibitory studies**					
	AChE (mg GALAE/g extract)^a^	BChE (mg GALAE/g extract)^a^	Tyrosinase (mgKAE/g extract)^a^	α-Amylase (mmolACAE/g extract)^a^	α-Glucosidase (mmolACAE/g extract)^a^	
	4.31 ± 0.00	6.25 ± 0.69	53.19 ± 0.40	0.55 ± 0.02	1.78 ± 0.01	

a Values expressed are means ± S.D. of three parallel measurements. HDEO: Hydro-distilled essential oil of fruit peels of *Citrus paradisi* var. *Foster*. TE: Trolox equivalent; EDTAE: EDTA equivalent. AChE: Acetylcholinesterase; BChE: Butyrylcholinesterase; GALAE: Galatamineequivalent; KAE: Kojic acid equivalent; ACAE: Acarbose equivalent; na: not active.

### 3.2 Enzyme inhibition activities of the HDEO

Fruit peel hydro-distilled essential oil (HDEO) of *C. paradisi* was also investigated for its enzyme inhibition potential against AChE, BChE, tyrosinase, α-amylase and α-glucosidase. It was weakly active against AChE and BChE, but showed potent tyrosinase inhibition with the value of 53.19 ± 0.40 mgKAE/g extract (Table 2). Literature reports also endorsed our results, since citrus essential oils have shown tyrosinase inhibitory activity [12,[67], [68], [69]]. Recently, Yang et al. have studied fruit peel essential oil of several citrus spices and found all of them as anti-melanogenesis, and suggested their use in cosmetics and pharmaceutics against skin hyperpigmentation [70]. It is also reported that limonene and terpineol are the most active component in citrus essential oils. In another report, da Silva et al. disclosed that eudesmol and caryophyllene are potential inhibitors of the enzyme tyrosinase, which they confirmed trough in vitro and in silico studies [71]. GCMS analysis of HDEO disclosed the presence of limonene as main component (∼54 %) as reported previously [72], along with eudesmol, caryophyllene, nootkatone as major ingredients and several other important components thus substantiate the worth of HDEO as potential ingredient in skin care products.

HDEO also exhibited moderate antidiabetic properties, since it exerted significant inhibitory activity against α-glucosidase (1.78 ± 0.01 mmolACAE/g extract) and weak activity (0.55 ± 0.02 mmolACAE/g extract) against α-amylase. Previously Dang et al. also studied α-glucosidase inhibitory activity of citrus fruit peel essential oils and found notable antidiabetic properties [73]. Oboh et al. also reported antidiabetic potential of essential oils from orange and lemon fruit peels [74]. In published data, α-glucosidase activities of citrus fruit peel essential oils are attributed to its terpene contents, since they exert blood glucose lowering effects [[75], [76], [77]] Guo et al. studied α-glucosidase inhibitory activities of a nootkatone derivative in *C. paradisi* fruit peel essential oil, and found to show antidiabetic properties [78]. The HDEO is also rich in monoterpenes and thus the α-glucosidase and α-amylase inhibitory activities are attributed to its monoterpene contents.

### 3.3 Post dock analysis

**Fig. 2:**
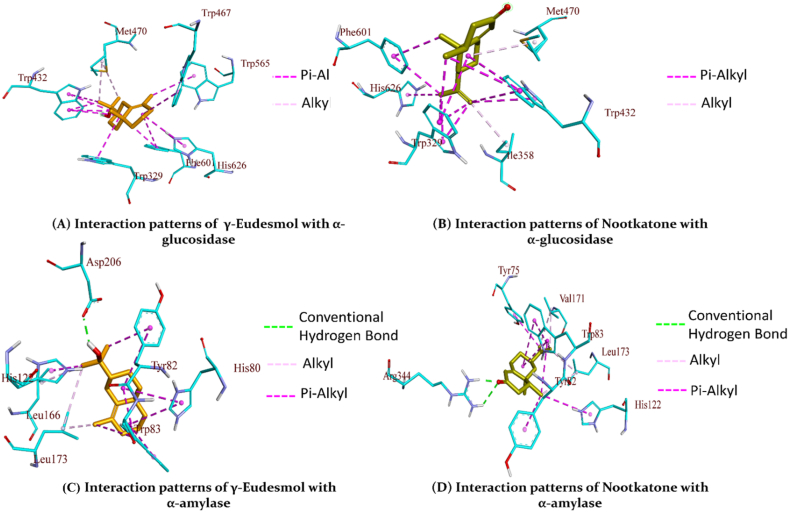
Binding interactions of compounds with α-glucosidase and α-amylase; A). Interaction patterns of γ-Eudesmol with α-glucosidase; B). Interaction patterns of Nootkatone with α-glucosidase; C). Interaction patterns of γ-Eudesmol with α-amylase; D). Interaction patterns of Nootkatone with α-amylase.

**Fig. 3:**
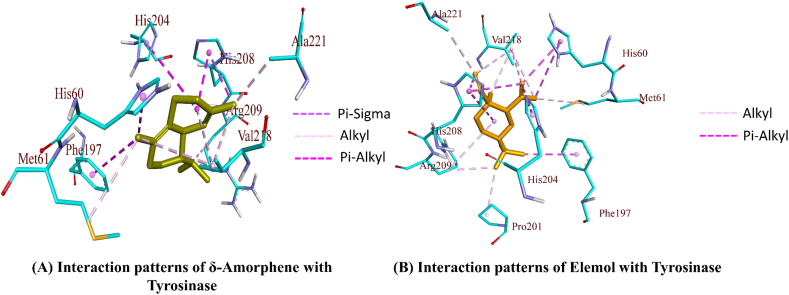
Binding interactions of compounds with tyrosinase; A). Interaction patterns of δ-Amorphene with tyrosinase; B). Interaction patterns of Elemol with tyrosinase.

The molecular interaction that transports the ligand from the protein surface to the active site indicates the significant affinities for the target proteins that were identified in docking studies. γ-Eudesmol, a sesquiterpenoid compound, was consistently shown to have lower binding energies for all three enzymes and have greater affinity towards α-amylase while nootkatone and caryophyllene showed lower binding affinity along with γ-Eudesmol towards α-glucosidase and tyrosinase respectively described in Table 3, Table 4. The post dock analysis revealed that γ-Eudesmol and nootkatone showed binding energies −8.70 kcal/mol and −7.20 kcal/mol for α-glucosidase, which is comparable to the binding affinity of acarbose −9.62 kcal/mol. The interaction patterns suggested that both compounds form a number of hydrophobic interactions while it fail to form any type of hydrogen bonds (Fig. 2 A-B). The interactions patterns suggest that as both compounds fail to depict hydrogen bonds, may be one of the reason to have lesser binding affinity than acarbose. Similar trend was also observed for α-amylase where acarbose formed nine hydrogen bond with an additional Pi-donor hydrogen bond contributing towards an overall binding affinity of −9.58 kcal/mol. The nootkatone formed two hydrogen bonds along with alkyl and pi-alkyl interactions and contributed towards binding affinity of −6.58 kcal/mol. The γ-Eudesmol formed same interactions as nootkatone and contributed binding affinity of −6.17 kcal/mol (Fig. 2C–D). The five compounds showed a significant binding affinity towards tyrosinase almost equal to kojic acid. The caryophyllene, γ-Eudesmol and nootkatone showed better binding affinity than rest of the two compounds. Although both compounds caryophyllene, and γ-Eudesmol formed only hydrophobic interactions but the number of interactions formed by these two compounds were enough to raise their binding affinity to −6.05 kcal/mol and −5.85 kcal/mol respectively (Fig. 3A–B). Table 2 summarizes the binding interaction patterns of compounds with binding affinity along with reference compound**s.**

**Table 3:** Binding free energy and inhibition constants of compounds α-glucosidase, α-amylase, and Tyrosinase.

Compounds	α-glucosidase		α-amylase		Tyrosinase	
	Free Energy of Binding kcal/mol	Estimated Inhibition Constant	Free Energy of Binding kcal/mol	Estimated Inhibition Constant	Free Energy of Binding kcal/mol	Estimated Inhibition Constant
aReference Compound	−9.62	88.89 nM	−9.58	95.58 nM	−4.07	1.04 mM
Caryophyllene	−6.99	7.53 μM	−5.83	53.42 μM	−6.05	36.70 μM
γ-Eudesmol	−8.70	417.58 nM	−6.17	30.16 μM	−5.85	51.85 μM
Limonene	−6.12	32.53 μM	−4.14	917.59 μM	−4.32	683.21 μM
Nootkatone	−7.20	5.32 μM	−6.58	14.92 μM	−5.67	69.32 μM
Terpineol	−6.04	37.26 μM	−4.56	452.98 μM	−4.66	386.57 μM
δ-Amorphene	−7.71	2.22 μM	−6.47	18.21 μM	−7.07	6.54 μM
Elemol	−6.96	7.90 μM	−5.75	61.25 μM	−6.14	31.59 μM
Di-iso-octyl phthalate	−6.59	14.81 μM	−5.27	137.41 μM	−4.72	347.40 μM

a For α-glucosidase and α-amylase, acarbose is used as reference compound. While kojic acid is used as reference for Tyrosinase.

**Table 4:** Details of interaction patterns of docked complex for Selected compounds.

*Ligands*	Bond Category	*Bond Distance*	*Bond type*	*Interactions*			
				*Residue Name and Groups*	*From* Chemistry	*Residue Name and Groups*	To Chemistry
Interaction Patterns for α–glucosidase							
Acarbose	Hydrogen Bond	2.77725	Conventional Hydrogen Bond	A:ARG629:HN	H-Donor	D:GLC1:O2	H-Acceptor
		2.80785		A:ASP630:HN		D:GLC1:O2	
		2.79736		D:GLC1:O6		D:AC13:O5	
		3.29623		D:GLC2:O3		A:ASP568:OD1	
		3.3492		D:AC13:O3		D:AC13:O6B	
		3.37223		D:AC13:N4A		A:ASP232:O	
		2.80319		D:AC13:O2B		A:ASP232:OD2	
	Hydrogen Bond	3.35883	Pi-Donor Hydrogen Bond	D:AC13:O3B	H-Donor	A:PHE476	Pi-Orbitals
γ-Eudesoml	Hydrophobic	5.49239	Alkyl	LIG0:C	Alkyl	A:MET470	Alkyl
		4.8416		LIG0:C		A:MET470	
	Hydrophobic	5.09268	Pi-Alkyl	A:TRP329	Pi-Orbitals	LIG0	Alkyl
		5.31521		A:TRP432		LIG0:C	
		4.34795		A:TRP432		LIG0:C	
		4.23027		A:TRP432		LIG0:C	
		5.20404		A:TRP432		LIG0	
		4.43833		A:TRP432		LIG0:C	
		5.10786		A:TRP467		LIG0	
		4.80578		A:TRP565		LIG0:C	
		5.24264		A:PHE601		LIG0	
		4.88464		A:PHE601		LIG0	
		5.01613		A:HIS626		LIG0	
Nootkatone	Hydrophobic	5.48322	Alkyl	A:MET470	Alkyl	LIG0	Alkyl
		4.32919		LIG0:C		A:ILE358	
	Hydrophobic	4.1586	Pi-Alkyl	A:TRP329	Pi-Orbitals	LIG0:C	Alkyl
		3.74465		A:TRP329		LIG0:C	
		5.33381		A:TRP329		LIG0	
		4.45396		A:TRP329		LIG0:C	
		4.46493		A:TRP329		LIG0:C	
		4.01912		A:TRP329		LIG0:C	
		4.98923		A:TRP432		LIG0:C	
		4.95507		A:TRP432		LIG0	
		5.16287		A:TRP432		LIG0:C	
		5.06696		A:TRP432		LIG0:C	
		4.54004		A:PHE601		LIG0:C	
		4.5383		A:PHE601		LIG0:C	
		4.6795		A:HIS626		LIG0:C	
Interaction Patterns for α–amylase							
Acarbose	Hydrogen Bond	2.00279	Conventional Hydrogen Bond	A:GLY167:HN	H-Donor	A:ABC479:O3H	H-Acceptor
		2.99957		A:ABC479:O3G		A:ABC479:O1L	
		2.6108		A:ABC479:O4G		A:ABC479:O3L	
		3.36955		A:ABC479:O2H		A:ABC479:O3I	
		2.74339		A:ABC479:O3I		A:ILE152:O	
		2.97893		A:ABC479:O2I		A:ILE152:O	
		3.31839		A:ABC479:O2I		A:GLN153:O	
		3.11009		A:ABC479:O2L		A:GLU230:OE2	
		2.80189		A:ABC479:O6I		A:ABC479:O6H	
	Hydrogen Bond	3.20313	Pi-Donor Hydrogen Bond	A:ABC479:O3K	H-Donor	A:TYR155	Pi-Orbitals
γ-Eudesoml	Hydrogen Bond	2.04215	Conventional Hydrogen Bond	LIG0:H	H-Donor	A:ASP206:OD2	H-Acceptor
	Hydrophobic	4.23028	Alkyl	LIG0:C	Alkyl	A:LEU166	Alkyl
		5.26636		LIG0:C		A:LEU173	
		3.9084		LIG0:C		A:LEU173	
	Hydrophobic	5.14759	Pi-Alkyl	A:HIS80	Pi-Orbitals	LIG0	Alkyl
		5.42681		A:HIS80		LIG0	
		4.48178		A:TYR82		LIG0:C	
		5.46758		A:TYR82		LIG0	
		4.87703		A:TRP83		LIG0	
		4.40036		A:TRP83		LIG0	
		5.21443		A:TRP83		LIG0:C	
		4.58881		A:TRP83		LIG0	
		4.29897		A:HIS122		LIG0:C	
Nootkatone	Hydrogen Bond	1.9922	Conventional Hydrogen Bond	A:ARG344:HH12	H-Donor	LIG0:O	H-Acceptor
		2.64594		A:ARG344:HH22		LIG0:O	
	Hydrophobic	4.99489	Alkyl	LIG0:C	Alkyl	A:LEU173	Alkyl
		3.68782		LIG0:C		A:VAL171	
		3.78241		LIG0:C		A:LEU173	
		4.43959		LIG0:C		A:VAL171	
	Hydrophobic	4.60171	Pi-Alkyl	A:TYR75	Pi-Orbitals	LIG0:C	Alkyl
		5.34175		A:TYR82		LIG0:C	
		4.98375		A:TRP83		LIG0	
		4.64208		A:TRP83		LIG0:C	
		4.33118		A:TRP83		LIG0:C	
		5.26202		A:TRP83		LIG0	
		4.95911		A:TRP83		LIG0:C	
		5.37695		A:HIS122		LIG0:C	
Interaction Patterns for Tyrosinase							
Kojic Acid	Hydrogen Bond	2.17753	Conventional Hydrogen Bond	A:ASN205:HD22	H-Donor	A:KOJ1351:O6	H-Acceptor
		3.20502		A:KOJ1351:O2		A:ASN205:O	
		2.83555		A:KOJ1351:O6		A:GLU195:OE1	
	Hydrogen Bond	3.31649	Carbon Hydrogen Bond	A:HIS204:CE1	H-Donor	A:KOJ1351:O5	H-Acceptor
		3.70276		A:HIS204:CE1		A:KOJ1351:O6	
δ-Amorphene	Hydrophobic	3.68229	Pi-Sigma	:UNN0:C	C–H	A:HIS208	Pi-Orbitals
	Hydrophobic	4.66568	Alkyl	A:VAL218	Alkyl	:UNN0	Alkyl
		3.37349		A:ALA221		:UNN0:C	
		4.76268		:UNN0:C		A:MET61	
		5.27111		:UNN0:C		A:VAL218	
		4.8625		:UNN0:C		A:VAL218	
		4.41771		:UNN0:C		A:ARG209	
	Hydrophobic	3.93042	Pi-Alkyl	A:HIS60	Pi-Orbitals	:UNN0:C	Alkyl
		5.46735		A:PHE197		:UNN0:C	
		5.20058		A:HIS204		:UNN0	
		3.55883		A:HIS208		:UNN0	
Elemol	Hydrophobic	5.30146	Alkyl	A:ARG209	Alkyl	:UNN0	Alkyl
		3.74544		A:ALA221		:UNN0:C	
		3.89686		:UNN0:C		A:PRO201	
		4.05458		:UNN0:C		A:ARG209	
		4.16395		:UNN0:C		A:VAL218	
		5.08068		:UNN0:C		A:MET61	
		4.7168		:UNN0:C		A:VAL218	
		4.5477		:UNN0:C		A:VAL218	
		4.58365		:UNN0:C		A:VAL218	
	Hydrophobic	4.81841	Pi-Alkyl	A:HIS60	Pi-Orbitals	:UNN0:C	Alkyl
		4.12758		A:HIS60		:UNN0:C	
		4.3964		A:PHE197		:UNN0:C	
		4.68953		A:HIS204		:UNN0:C	
		5.43544		A:HIS208		:UNN0	
		4.66318		A:HIS208		:UNN0:C	
		3.68856		A:HIS208		:UNN0:C

## 4 Conclusion

*C. paradisi* var. Foster fruit peel hydro-distilled essential oil (HDEO) comprised mostly of limonene, nootkatone, γ-Eudesoml and caryophyllene. HDEO also exhibited moderate to good antioxidant and antidiabetic activities in various assays, which make this oil important component in several skin and health care products or in nutraceuticals. Another worthy feature of HDEO is the inhibition of tyrosinase enzyme. Since limonene, terpineol, eudesmol and caryophyllene have already shown tyrosinase inhibitory or anti-melanogenesis properties, the HDEO rich in these components can be a potential ingredient in skin care products for hyperpigmentation and melanogenesis. Further docking studies confirm that caryophyllene, γ-eudesoml, and nootkatone bind well to α-glucosidase and α-amylase, but all investigated compounds had a greater binding affinity for tyrosinase. In local market, after the extraction of juice, the peels of *C. paradisi* are thrown as waste, therefore, it is suggested that essential oil of *C. paradisi* fruit peels if added to skin care products or other formulations may not only act as fragrance but also are potential active ingredient; even addition of dried powdered peels of *C. paradisi* var. Foster to skin cleansing products will increase their anti-hyperpigmentation and anti-melanogenesis effects and may reduce to decrease the cost of the products. Browning of stored food is a big problem in food industry, whereas, citrus peel essential oils increase the shelf-life of certain fruits, and thus played important role in food preservation. Keeping in view these facts and anti-tyrosinase activity of HDEO, it is suggested that essential oil of fruit peels of *C. paradisi* var. Foster can also be used as food preservative.

## Funding

The authors would like to extend their sincere appreciation to the Researchers Supporting Project Number (RSP2024R134), 10.13039/501100002383King Saud University, Riyadh, Saudi Arabia.

## CRediT authorship contribution statement

**Rameen Sajid:** Investigation. **Zaheer Abbas:** Writing – review & editing. **Mamona Nazir:** Formal analysis. **Muhammad Saleem:** Writing – original draft, Supervision, Conceptualization. **Naheed Riaz:** Supervision, Investigation. **Muhammad Imran Tousif:** Writing – review & editing, Writing – original draft, Investigation, Formal analysis. **Saba Tauseef:** Writing – original draft, Formal analysis. **Gokhan Zengin:** Writing – review & editing, Writing – original draft, Investigation, Formal analysis. **Abdullahi Ibrahim Uba:** Investigation, Formal analysis. **Abdullah Ijaz Hussain:** Investigation, Formal analysis. **Muhammad Shaiq Ali:** Investigation, Formal analysis. **Abeer Hashem:** Writing – review & editing, Software, Funding acquisition. **Khalid F.Almutairi:** writing- review & editing, funding acquistion. **Graciela Dolores Avila-Quezada:** Avila-Quezada, Funding acquisition, Formal analysis. **Elsayed Fathi Abd_Allah:** Funding acquisition, Formal analysis.

## Declaration of competing interest

The authors declare that they have no known competing financial interests or personal relationships that could have appeared to influence the work reported in this paper.
