# Supplementary material for: Valorization of hydro-distillate of fruit peels of Citrus paradisi macfad. Cultivar. Foster: Chemical profiling, antioxidant evaluation and in vitro and in silico enzyme inhibition studies
Source: Heliyon. 2024 Aug 21;10(17):e36226. doi: 10.1016/j.heliyon.2024.e36226 (PMC11400606; doi:10.1016/j.heliyon.2024.e36226)
Supplement: Multimedia component 1 [file mmc1.docx]

**Valorization of hydro-distillate of fruit peels of *Citrus paradisi* Macfad. cultivar. Foster: Chemical profiling, antioxidant evaluation and *in vitro* and in silico enzyme inhibition studies**

Rameen Sajid^1^, Zaheer Abbas^2^, Mamona Nazir^3^, Muhammad Saleem^1*^, Naheed Riaz^1^, Muhammad Imran Tousif^4*^, Saba Tauseef^5^, Gokhan Zengin^6*^, Abdullahi Ibrahim Uba^7^, Abdullah Ijaz Hussain^8^, Muhammad Shaiq Ali^9^, Abeer Hashem^10^, Khalid F. Almutairi^11^, Graciela Dolores Avila-Quezada^12^, Elsayed Fathi Abd_Allah^11^

^1^Institute of Chemistry, Baghdad-ul-Jadeed Campus, The Islamia University of Bahawalpur, 63100, Bahawalpur, Pakistan

^2^Department of Botany, Division of Science and Technology, University of Education Lahore, Pakistan

^3^Department of Chemistry, Government Sadiq College Women University Bahawalpur, 63100 Bahawalpur, Pakistan

^4^Department of Chemistry, Division of Science and Technology, University of Education Lahore, Pakistan

^5^Dr. Panjwani Center for Molecular Medicine and Drug Research., International Center for Chemical and Biological Sciences, University of Karachi, Karachi, Pakistan

^6^Department of Biology, Science Faculty, Selcuk University, 42130 Konya, Turkey

^7^Department of Molecular Biology and Genetics, Istanbul AREL University, 34537 Istanbul, Türkiye

^8^Department of Chemistry, GC University Faisalabad, Pakistan

^9^International Center of Chemical and Biological Sciences, University of Karachi, 75270 Karachi, Pakistan

^10^ Botany and Microbiology Department, College of Science, King Saud University, P.O. Box. 2460, Riyadh 11451, Saudi Arabia (A.H., habeer@ksu.edu.sa).

^11^Facultad de Ciencias Agrotecnológicas, Universidad Autónoma de Chihuahua, 31350, Chihuahua, Chihuahua, México. (GDAQ., gdavila@uach.mx).

^12^Plant Production Department, College of Food and Agricultural Sciences, King Saud University, P.O. Box. 2460, Riyadh 11451, Saudi Arabia (K.F.A., [almutairik@ksu.edu.sa](mailto:almutairik@ksu.edu.sa); E.F.A., eabdallah@ksu.edu.sa).

***Corresponding author**: ^*^Corresponding author: Prof. M. Saleem ([m.saleem@iub.edu.pk](mailto:m.saleem@iub.edu.pk)), Muhammad Imran Tousif ([Imran.tousif@ue.edu.pk](mailto:Imran.tousif@ue.edu.pk)), Prof. Dr. Gokhan Zengin ([gokhanzengin@selcuk.edu.tr](mailto:gokhanzengin@selcuk.edu.tr))

**Identification of the components in essential oils (HDEO) of fruit peels of *Citrus paradisi* Macfad. cultivar. Foster**

GCMS chromatogram displayed several components (Figure 1), however, 12 of them were identified.

**Identification of Limonene**

70 eV of ionization energy was employed and obtained mass spectrum (Figure 2) to analyze components present in peels essential oil of *C. paradisi*. The first peak observed at retention time 13.33 min indicates the limonene constituent implying its molecular formula C_10_H_16_. The peak observed at *m/z* 136 was molecular ion peak and its base peak was observed at *m/z* 68, which was attributed to a fragment ion C_5_H_8_^+^ formed by the dissociation of parent ion. When parent ion dissociate by loss of CH_3_^•^ then fragment ion (M-CH_3_)^+^ at *m/z* 121 was formed. The peak observed at *m/z* 107 was due to formation of fragment ion C_8_H_11_^+^ by means of loss of ethyl radical from molecular ion (Abd El-Kareem et al., 2016). The two peaks observed at *m/z* 93 and 79 were due to formation of fragment ions C_7_H_9_^+^ and C_6_H_7_^+^, respectively. Other fragment ions of *m/z* 53 and 39 were also observed. The fragmentation pathway of limonene has been shown in scheme 1.

**Figure 1:** Chromatogram of *Citrus paradisi* cultivar Foster peel essential oil

**Figure 2:** Mass spectrum of limonene

**Scheme 1:** Fragmentation pathway of limonene

**Identification of α-Terpineol**

The peak at retention time 17.67 min indicates the presence of α-terpineol component implying its molecular formula C_10_H_18_O. The most intense peak at *m/z* 59 was due to fragment ion C_3_H_7_O^+^ formed by the dissociation of parent ion. When molecular ion dissociates to form fragment ion (M−CH_3_−H_2_O) ^+^, then the peak observed at *m/z* 121. Other peak at *m/z* 93 was due to formation of fragment ion C_7_H_9_^+^. The fragment ion peaks at *m/z* 81 and 43 were also observed (Figure 3). Its fragmentation pattern was shown in scheme 2.

**Figure 3:** Mass spectrum of α-terpineol

**Scheme 2:** Fragmentation pathway of α-terpineol

**Identification of Caryophyllene**

The peak at retention time 23.29 min indicated the caryophyllene constituent; its molecular formula C_15_H_24_ was determined due to molecular ion at *m/z* 204. Their fragment ions peaks were observed at *m/z* 189, 175, 161, 147, 133, 120, 105, 91, 79, 69, 55, 44 and 41. The loss of methyl group from molecular ion resulted in fragment ion at m/z 189 (M-CH_3_)^+^, while when C_2_H_5_ loss then peak observed at *m/z* 175 to form ion (M-C_2_H_5_) ^+^ with low relative intensity (Yahaya et al., 2019)The two peaks appeared at *m/z* 161 and 147 were due to fragment ions C_12_H_17_ ^+^ and C_11_H_15_ ^+^ by the loss of C_3_H_7_ and C_4_H_9_ group from parent ion, as shown in scheme 6. The parent ion dissociates and formed fragment ions C_10_H_13_^+^ and C_8_H_9_^+^ at m/z 133 and 105 (Figure 7).

**Figure 7:** Mass spectrum of caryophyllene

**Scheme 6:** Fragmentation pathway of caryophyllene

**Identification of δ-Amorphene**

The peak at retention time 25.39 min indicated the presence of δ-amorphene, whose molecular formula as C_15_H_24_ was established due to the heaviest peak in mass spectrum (Figure 9) at *m/z* 204. The peak observed at *m/z* 189 was due to formation of fragment ion C_14_H_21_^+^ by means of loss of methyl radical from molecular ion. The intense peak was observed at *m/z* 161 by dissociating parent ion to form the main fragment ion (M-C_3_H_7_)^+^ by means of loss of C_3_H_7_ radical. The peak appeared at *m/z* 134 was due to fragment ion C_10_H_14_^+^ resulted from removal of C_5_H_10_ group. Other fragment ions at *m/z* 143, 129, 119, 105, 91, 77, 67, 55, 44 and 41 were also observed. Its fragmentation pattern was shown in scheme 8.

**Figure 9:** Mass spectrum of δ-amorphene

**Scheme 8:** Fragmentation pathway of δ-amorphene

**Identification of Elemol**

The peak at retention time 26.00 min indicated the constituent as elemol component implying its molecular formula C_15_H_26_O. Their fragment ions peaks were observed at *m/z* 189, 161, 147, 134, 119, 107, 93, 79, 67, 59, 55 and 43 (Figure 10). Parent ion dissociates to produce fragment ion C_14_H_21_^+^ at *m/z* 189 by the loss of methyl and water molecule from it. Further fragmentation of C_14_H_21_^+^ produced fragment ion C_12_H_17_^+^ at *m/z* 161 by means of loss of C_2_H_4_ molecule. The base peak was observed at *m/z* 59 because of the formation of fragment ion C_3_H_7_O^+^. Fragment ion C_12_H_17_^+^ undergo fragmentation to produce C_10_H_14_ ^+^ at *m/z* 134 by the loss of C_2_H_3_ molecule, as shown in scheme 9.

**Figure 10:** Mass spectrum of elemol

**Scheme 9:** Fragmentation pathway of elemol

**Identification of γ-Eudesmol**

The peak in chromatogram appeared at 28.40 min was found to be due to the component γ-eudesmol. The mass spectrum displayed molecular ion at *m/z* 222 leading to the molecular formula C_15_H_26_O. The peak appeared at *m/z* 204 was due to fragment ion C_15_H_24_^+^ by the loss of water molecule from molecular ion. Further fragmentation of C_15_H_24_^+^ ion produced two intense peaks at *m/z* 189 and 161 due to ions (M-H_2_O-CH_3_) ^+^ and (M-H_2_O-C_3_H_7_)^+^ respectively. Further fragmentation of C_12_H_17_^+^ at *m/z* 161 produced fragment ion C_10_H_13_^+^ at *m/z* 133 by the loss of C_2_H_4_ molecule from it. The fragment ion of *m/z* 133 undergoes fragmentation to produce ion C_7_H_7_^+^ at m/z 91. Other fragment ions of *m/z* 175, 147, 119, 107, 79, 67, 59, 55 and 41 were also observed in the mass spectrum (Figure 11). Main fragmentation pattern of γ-eudesmol was shown in scheme 10.

**Figure 11:** Mass spectrum of γ-eudesmol

**Scheme 10:** Fragmentation pathway of γ-eudesmol

**Identification of Nootkatone**

The constituent nootkatone was eluted at retention time 35.79 min, the molecular ion peak at *m/z* 218 in the mass spectrum (Figure 12) depicted the molecular formula C_15_H_22_O. The two peaks appeared at *m/z* 203 and 147 were due to fragment ions C_14_H_19_O^+^ and C_10_H_11_O^+^ by the loss of methyl and C_5_H_11_ from parent ion. Fragment ion C_3_H_5_^+^ formed by dissociating parent ion and as a result the intense peak was observed at *m/z* 41. Removal of CO molecule from parent ion, form the fragment ion (M-CO)^+^ at *m/z* 190. Other fragment ions of *m/z* 175, 161, 133, 121, 105, 91, 79, 73, 67, and 53 were also observed. These fragment ions that were formed by the dissociation of parent ion, shown in scheme 11.

**Figure 12:** Mass spectrum of nootkatone

**Scheme 11:** Fragmentation pathway of nootkatone

**Identification of Decanal**

The peak at retention time 17.89 min was identified due to decanal and its molecular formula was found to be C_10_H_20_O. The intense peaks at *m/z* 43 and 57 were due to fragment ions C_2_H_3_O^+^ and C_3_H_5_O^+^ formed through dissociation of parent ion. The peak due to fragment ion C_8_H_16_^+^ appeared at *m/z* 112 by the loss of water and ethyne molecule from molecular ion as shown in scheme 3. Other fragment ions peaks were observed at *m/z* 95 and 82 (Figure 4).

**Figure 4:** Mass spectrum of decanal

**Scheme 3:** Fragmentation pathway of decanal

**Identification of 1-Decanol**

At retention time 19.46 min, the peak observed indicated presence of 1-decanol constituent implying its molecular formula C_10_H_22_O. The fragment peaks at *m/z* 43 and 41 were due to fragment ions C_3_H_7_^+^ and C_3_H_5_^+^ formed through dissociation of parent ion. The loss of water and ethene molecule from molecular ion produces C_8_H_16_^+^ at *m/z* 112. Fragmentation of C_8_H_16_^+^ ion produce peaks at *m/z* 69 and 55 because of formation of fragment ions C_5_H_9_^+^ and C_4_H_7_^+^ respectively. Further fragmentation of fragment ion C_8_H_16_^+^ produced two peaks at *m/z* 97 and 83 by the loss of methyl and ethyl molecule from it. Other fragment ion peaks were observed at *m/z* 111 and 53 (Figure 5). Main fragmentation pattern of 1-decanol was shown in scheme 4.

**Figure 5:** Mass spectrum of 1-decanol

**Scheme 4:** Fragmentation pathway of 1-decanol

**Identification of** **n-Decanoic acid**

The peak observed at retention time 21.50 min was attested for n-decanoic acid implying its molecular formula C_10_H_20_O_2_, since the molecular ion peak was observed at *m/z* 172. When parent ion dissociates to form fragment ion C_3_H_5_O_2_^+^, the peak observed at *m/z* 73. Other peak at *m/z* 129 was due to the formation of fragment ion C_7_H_11_O_2_^+^. The dissociation of parent ion through Mclafferty rearrangement to form fragment ion C_2_H_4_O_2_^+^ by means of loss of C_8_H_16_ group, as a result intense peak was observed at *m/z* 60. Other fragment ions of *m/z* 83, 69 and 43 were also observed (Figure 6). The fragmentation pathway of n-decanoic acid was shown in scheme 5.

**Figure 6:** Mass spectrum of n-decanoic acid

**Scheme 5:** Fragmentation pathway of n-decanoic acid

**Identification of 2,4-di-tert-butyl phenol**

The peak in chromatogram appeared at 24.89 min was attributed to 2,4-ditertiarybutyl phenol; its molecular ion at *m/z* 206 suggested the molecular formula as C_14_H_22_O. Fragment ion C_13_H_19_O^+^ formed by dissociating parent ion and as a result the most intense peak was observed at *m/z* 191, which indicated loss of methyl group from parent ion. When molecular ion dissociate by loss of C_10_H_13_O radical, it form fragment ion (M-C_10_H_13_O)^+^ at *m/z* 57. The peak at *m/z* 91was due to fragment ion C_7_H_7_^+^ has low relative intensity, formed by the dissociation of parent ion. Other fragment ions at *m/z* 175, 163, 147, 128, 107, 77, 44 and 41 were also observed (Figure 8). Main fragmentation pattern of 2,4-di-tert-butyl phenol was shown in scheme 7.

**Figure 8:** Mass spectrum of 2,4-di-tert-butyl phenol

**Scheme 7:** Fragmentation pathway of 2, 4-di-tert-butyl phenol

**Identification of diisooctyl phthalate**

The peak at retention time 64.46 min indicated the presence of diisooctyl phthalate constituent suggesting its molecular formula C_24_H_38_O_4_. The intense peak observed at *m/z* 149 was due to the formation of fragment ion C_5_H_8_O_3_^+^ by dissociating parent ion. The peak at *m/z* 132 was due to fragment ion C_8_H_4_O_2_^+^ (Yin *et al*., 2014). The peaks observed at *m/z* 113 and 43 were due to fragment ions C_8_H_17_^+^ and C_3_H_7_^+^. Other fragment ions at *m/z* 279, 167, 83, 71 and 57 were also observed in the mass spectrum (Figure 13). Main fragmentation pattern of diisooctyl phthalate was shown in scheme 12.

**Figure 13:** Mass spectrum of diisooctyl phthalate

**Scheme 12:** Fragmentation pathway of diisooctyl phthalate

**References**

Abd El-Kareem, M.S., Rabbih, M.A.E.F., Selim, E.T.M., Elsherbiny, E.A.E.-m., and El-Khateeb, A.Y. (2016). Application of GC/EIMS in combination with semi-empirical calculations for identification and investigation of some volatile components in basil essential oil. International Journal of Analytical Mass Spectrometry and Chromatography *4*, 14-25.

Yahaya, M.F., Yelwa, J.M., Abdullahi, S., Umar, J.B., Abubakar, A.M., and Babakura, M. (2019). Chemical compositions, FTIR and larvicidal activity of essential oils extracted from aromatic plants. Eur Sci J ESJ *15*, 110.
